# Exploring the geochemical distribution of organic carbon in early land plants: a novel approach

**DOI:** 10.1098/rstb.2016.0499

**Published:** 2017-12-18

**Authors:** Geoffrey D. Abbott, Ian W. Fletcher, Sabrina Tardio, Ethan Hack

**Affiliations:** 1 School of Natural and Environmental Sciences, Newcastle University, Drummond Building, Newcastle upon Tyne NE1 7RU, UK; 2 National EPSRC XPS Users’ Service (NEXUS), School of Engineering, Newcastle University, Newcastle upon Tyne NE1 7RU, UK; 3 School of Natural and Environmental Sciences, Newcastle University, Ridley Building, Newcastle upon Tyne NE1 7RU, UK

**Keywords:** time-of-flight secondary ion mass spectrometry, *Rhynia gwynne-vaughanii*, X-ray photoelectron spectroscopy, ‘chemical maps’, terrestrialization

## Abstract

Terrestrialization depended on the evolution of biosynthetic pathways for biopolymers including lignin, cutin and suberin, which were concentrated in specific tissues, layers or organs such as the xylem, cuticle and roots on the submillimetre scale. However, it is often difficult, or even impossible especially for individual cells, to resolve the biomolecular composition of the different components of fossil plants on such a scale using the well-established coupled techniques of gas chromatography/mass spectrometry and liquid chromatography/mass spectrometry. Here, we report the application of techniques for surface analysis to investigate the composition of *Rhynia gwynne-vaughanii*. X-ray photoelectron spectroscopy of two different spots (both 300 µm × 600 µm) confirmed the presence of carbon. Time-of-flight secondary ion mass spectrometry (ToF-SIMS) revealed ‘chemical maps’ (imaging mode with 300 nm resolution) of aliphatic and aromatic carbon in the intact fossil that correlate with the vascular structures observed in high-resolution optical images. This study shows that imaging ToF-SIMS has value for determining the location of the molecular components of fossil embryophytes while retaining structural information that will help elucidate how terrestrialization shaped the early evolution of land plant cell wall biochemistry.

This article is part of a discussion meeting issue ‘The Rhynie cherts: our earliest terrestrial ecosystem revisited’.

## 1. Introduction

The colonization of land by plants in the mid-Palaeozoic is recorded in fossil assemblages that possess diverse morphology rarely seen in extant organisms [1] and often lack some of the organs characteristic of plants [2]. Such limitations hamper identification even to phylum level, although there is growing evidence that early terrestrial vegetation included vascular plants, cyanobacteria, algae, basal bryophytes, fungi and lichens [3]. The biosyntheses of lignin, cutin and suberin contribute to water transport and permeability control in vascular plants, and these were all key to the survival and eventual proliferation of land plants in terrestrial depositional environments [4–6]. These different biopolymers are associated with specific tissues, layers or organs, e.g. lignin penetrates the cellulosic framework in xylem [4], whereas cutin and suberin provide barrier functions associated with the epidermis, endodermis and periderm [6]. The cuticle forms the protective layer outside the epidermis of most aerial vascular plant organs, and is composed mainly of cutin, whereas the stems of woody plants have a periderm with suberin as an important component [5]. Taphonomic and diagenetic processes act on the plant remains both before and after burial in the sedimentary column. These result in chemical alteration of such polymers. However, if the decay processes can be inhibited (e.g. through exclusion of oxygen or permineralization), then there may be preservation of at least some of the fossilized organic carbon [7]. Fossilization of plant remains involves one of four processes, namely coalification [8], permineralization [9], mummification [10] or charcoalification [11]. Mummification is the least understood of these processes and under exceptional circumstances preserves the original organic carbon with minimal alteration [12], in which case lignin phenols can be identified unequivocally with the addition of ^13^C-labelled tetramethylammonium hydroxide [13]. Coalification is where the wood is transformed into peat and then into soft brown coal by both aerobic and anaerobic biochemical processes, and then with further increase in the coalification rank to hard coal and finally graphite [9]. Primary and secondary products of lignin phenols are often present, namely the catechols and the alkylbenzenes that are formed via the chemical reactions of demethylation and dehydroxylation, which can also be simulated in the laboratory [14]. The organic carbon is preserved in the cell walls of a permineralization, but the original pore spaces are filled with minerals (silica in the case of the Rhynie chert) [9]. The challenge is now to identify the residues of these biopolymers in fossilized plants and their constituent tissues and organs—this requires a spatially resolved method to identify the degradation products of plant biopolymers and other natural products. To enhance the spatial resolution of our analytical procedures, we have explored the application of both X-ray photoelectron spectroscopy (XPS) and time-of-flight secondary ion mass spectrometry (ToF-SIMS) on a specimen of *Rhynia gwynne-vaughanii* from the Rhynie chert to obtain accurate mass spectral information from micrometre-scale analysis areas on specific components of whole fossil plants without the need for milling and subsequent solvent extraction [15].

## 2. Material and methods

### (a) Material

**Figure 1.**
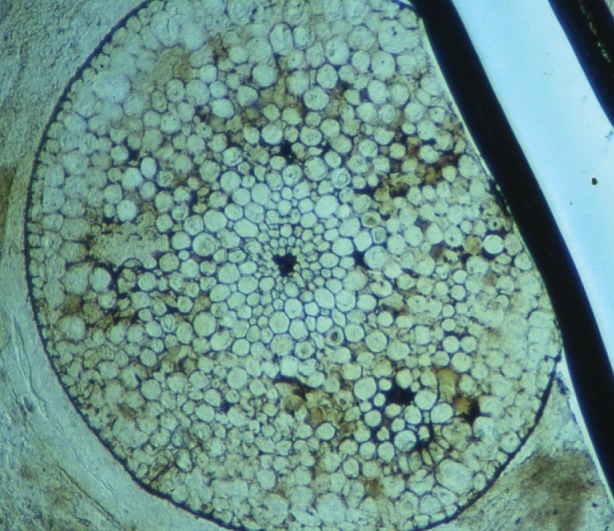
Optical image of *R. gwynne-vaughanii* specimen.

*R. gwynne-vaughanii* fossils (figure 1) were identified in a large sawn fragment of the Rhynie chert (about 5 × 5 cm).

Any potential post-depositional ingress was removed from the large fragment of the chert, using Soxhlet extraction with a mixture of dichloromethane/methanol (93 : 7; v/v) for 72 h [16]. XPS analysis both before and after argon gas cluster ion beam etching (at different energies) on the extracted fossil specimen revealed that there was no remaining contamination.

### (b) X-ray photoelectron spectroscopy

One specific fossil (approx. 2.4 mm in diameter) of *R. gwynne-vaughanii* was analysed by XPS in a Theta Probe instrument (Thermo Scientific, East Grinstead, UK). Spectra were acquired using a monochromatic Al Kα X-ray source with an output energy of 1486.6 eV and a spot size of 300 × 600 µm. Two spots were analysed. A dwell time of 50 ms was used for survey spectra and of 100 ms for high-resolution spectra. Surface charge compensation was carried out using a low-energy electron flood gun. Survey spectra were obtained with a step size equal to 1.0 eV and a pass energy of 200 eV, while high-resolution spectra were obtained with 0.4 eV step size and 40 eV pass energy. Data interpretation was carried out by means of the XPS manufacturer software Avantage v. 5.6925. This was used in order to determine the chemical composition of the surface of the samples as well as to obtain information on the chemical states of elements of interest. Peaks were fitted with Gaussian (70%)–Lorentzian (30%) components and quantified using relative sensitivity factors (Scofield) [17]. Shirley background subtraction was used before the peak fitting [18].

### (c) Time-of-flight secondary ion mass spectrometry

**Figure 2.**
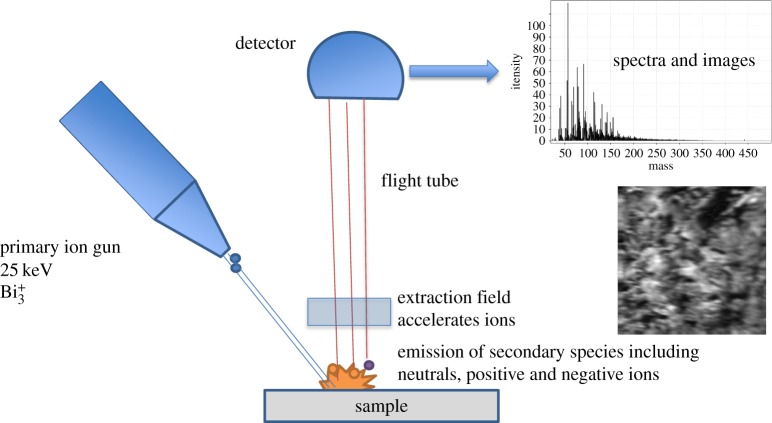
Schematic view of ToF-SIMS spectrometer.

The fragment of chert containing the *R. gwynne-vaughanii* was mounted directly onto a sample holder using stainless steel screws and clips for ToF-SIMS analysis. Static SIMS analyses were carried out using an ION-TOF ‘TOF-SIMS IV-200’ instrument (ION-TOF GmbH, Münster, Germany) of single-stage reflectron design [19]. Figure 2 shows a schematic illustration of the ION-TOF ‘TOF-SIMS IV-200’ instrument. Positive ion spectra and images of the sample were obtained using a Bi_3_^+^ focused liquid metal ion gun at 25 keV energy, incident at 45° to the surface normal and operated in ‘bunched’ mode for high mass resolution. This mode used 20 ns wide ion pulses at 10 kHz repetition rate. Charge compensation was effected by low-energy (approx. 20 eV) electrons provided by a flood gun. The total ion dose density was less than 1 × 10^16^ ions m^−2^. The topography of the sample surface and the ion gun mode of operation limited the mass resolution in this work to approximately *m*/Δ*m* = 2000. The spatial resolution was limited by the primary ion beam diameter to approximately 4 µm.

Positive and negative ion static SIMS spectra and images were recorded from the outermost approximately 1 nm of the sample surface at room temperature. Raw data containing the secondary ions recorded at each pixel were acquired with a 256 × 256 pixel raster and a field of view of 500 µm × 500 µm. Four adjacent 500 µm × 500 µm analysis areas were mapped starting at the approximate centre of the specimen and then moving the sample radially outward by 500 µm each time. The third area included the epidermis of the *R. gwynne-vaughanii* and some matrix material, while the final area included only the matrix for comparison.

## 3. Results

### (a) X-ray photoelectron spectroscopic analysis

**Figure 3.**
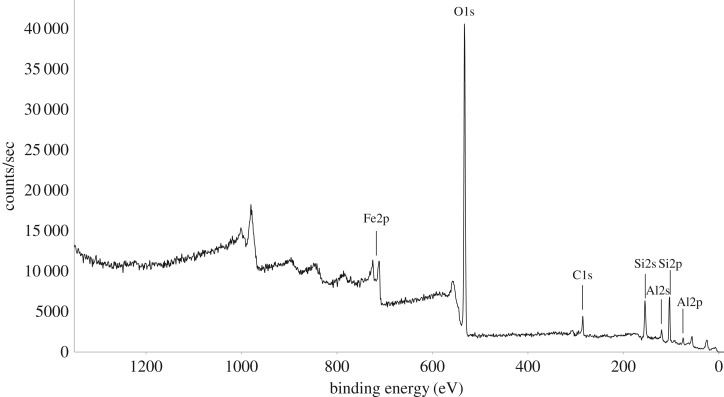
XPS survey spectrum of *R. gwynne-vaughanii.*

XPS spectra of *R. gwynne-vaughanii* reveal the presence of the elements oxygen, silicon, carbon, iron and aluminium (figure 3). Table 1 shows their surface concentrations at two spots on the fossil with a spot size of 300 × 600 µm. The two spots have a similar elemental composition which indicates the extent of mineralization with a relatively low carbon content.

**Table 1.** Percentage chemical composition, as atomic percentage, obtained from XPS high-resolution spectra on the two spots analysed on the specimen of *R. gwynne-vaughanii*.

element	O 1s	Si 2p	C 1s	Fe 2p	Al 2p
spot 1	63.7	25.0	5.8	2.8	2.7
spot 2	61.8	19.4	8.8	5.4	4.6

**Figure 4.**
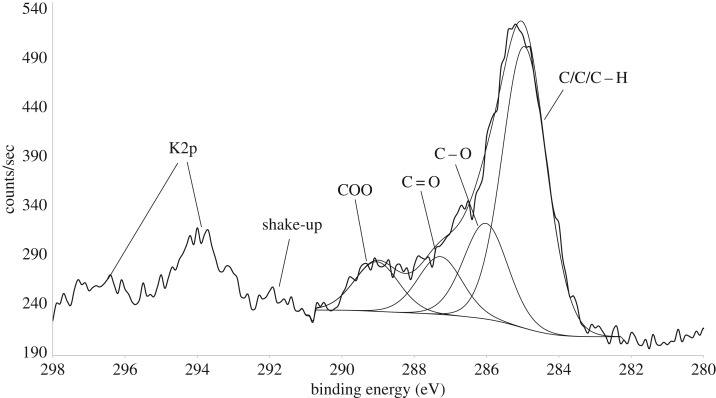
XPS C1s high-resolution fitted peaks for *R. gwynne-vaughanii.*

High-resolution spectra of O 1s, Si 2p, C 1s and Al 2p were also recorded. The C 1s high-resolution spectrum of *R. gwynne-vaughanii* is shown in figure 4. This spectrum is composed of overlapping peaks that can be deconvoluted into four components as assigned in figure 4 and table 2 [20]. The most intense peak at about 285.0 eV is consistent with *sp^3^* and *sp^2^* hybridized aliphatic/aromatic carbon bonded to only carbon or hydrogen. Additionally, there are peaks assigned to carbon single-bonded to oxygen (C–O at 286.1 eV), carbon double-bonded to oxygen (C=O at 287.3 eV) and carbon both single- and double-bonded to oxygen (O–C=O at 289.1 eV). The relative percentage abundance of the C 1s components is presented in table 2. Figure 4 also indicates a broad peak at approximately 292 eV which is assigned to a π–π* shake-up satellite, suggesting the presence of aromatic carbon or other conjugated unsaturated carbon moieties. Traces of potassium were also detected in the C1s high-resolution scan region (because the binding energy of the K 2p electron is close to that of the C 1s) (figure 4).

**Table 2.** Percentage of the total carbon present as C–C/C–H, C–O, C=O and O–C=O on the specimen of *R. gwynne-vaughanii*.

	percentage of total carbon present as:			
functionality	C–C/C–H	C–O	C=O	O–C=O
spot 1	67	18	8	8
spot 2	60	17	11	13

### (b) Time-of-flight secondary ion mass spectrometric analysis

**Figure 5.**
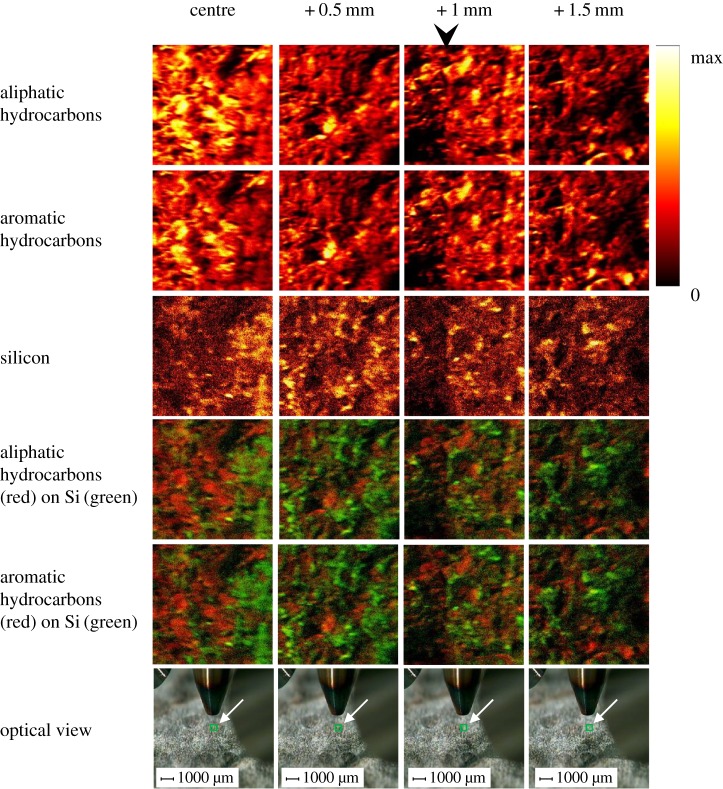
Secondary ion images of *R. gwynne-vaughanii* specimen. The epidermal region of the *R. gwynne-vaughanii* is indicated by the black arrowhead. The optical view was recorded from the instrument camera during the analysis. The green square at the approximate centre of each optical image (indicated by white arrow) represents the actual analysis area. The associated dark grey, approximately circular, area is the stem cross-section.

The results of the ToF-SIMS analysis are shown in figure 5. The aliphatic carbon and aromatic carbon ‘chemical maps’ were created from the sum of the individual maps due to the secondary fragment ions shown in table 3.

**Table 3.** Positive ions—assignment and accurate masses (*m/z)* abridged to two decimal places.

aliphatic hydrocarbon		aromatic hydrocarbon	
C_2_H_5_^+^	29.04	C_6_H_5_^+^	77.04
C_3_H_5_^+^	41.04	C_7_H_7_^+^	91.06
C_4_H_9_^+^	57.07	C_8_H_9_^+^	105.08
C_5_H_9_^+^	69.08	C_9_H_7_^+^	115.06
C_6_H_11_^+^	83.09	C_10_H_8_^+^	128.06
C_7_H_13_^+^	97.11	C_11_H_9_^+^	141.08
C_8_H_15_^+^	111.13	C_12_H_8_^+^	152.06
C_9_H_17_^+^	125.13	C_13_H_9_^+^	165.07
		C_14_H_10_^+^	178.07
^28^Si^+^	27.98	C_15_H_11_^+^	191.08

The Si map was created from the ^28^Si^+^ secondary ion alone. The individual secondary ion maps were regenerated from the raw dataset after recalibration of the mass scale. The mass resolution obtained was easily sufficient to enable selection of discrete peak areas for the specified ions. The left-hand column of maps and images was recorded from the approximate centre of the *R. gwynne-vaughanii*. The third column included the epidermis, as indicated by the arrow in figure 5, and some of the surrounding matrix material. The fourth column consisted entirely of matrix material. The aliphatic and aromatic hydrocarbon species detected showed similar distributions so that the aliphatic/aromatic ratio did not vary greatly across the specimen. Figure 5 shows that there is a region with relatively high content of both aliphatic and aromatic carbon near the centre of the stem, and a relatively depleted region in the outermost cortex. Both aliphatic and aromatic carbon are detectable in the matrix surrounding the stem as well. The spatial distribution of both types also appears to be somewhat negatively correlated with the distribution of ^28^Si^+^.

## 4. Discussion

The ability to characterize biomolecules and their taphonomic/diagenetic products in specific tissues, layers or organs such as the xylem, cuticle and roots on the submillimetre scale has the potential to increase our understanding of the palaeobiochemistry of early land plants. In this study, we describe a novel analytical approach to discriminate different spatial positions within *R. gwynne-vaughanii* in the Rhynie chert.

Commonly, the analytical approach has been firstly to mill or powder the specimen in gram amounts [16,21–24]. Then the powdered fossil is extracted with a dichloromethane/methanol solvent mixture and following chromatographic fractionation, the biomarkers are analysed using either combined gas chromatography/mass spectrometry (GC/MS) or high-performance liquid chromatography/mass spectrometry. Finally, the molecular characterization of the organic-insoluble residue from the solvent extraction process is commonly carried out using one or more of flash pyrolysis/GC/MS, FTIR and ^13^C solid-state NMR [16]. The same approach has been used to identify intracrystalline lipids in brachiopod shells [25]. Reliable detection of biomarkers typically requires centimetre-scale sized samples and the milling process will inevitably mix different components of the plant fossil together. This will remove all of the distributional information on the organic carbon in the fossil samples [15]. Alternatively, Raman spectroscopy has been used to identify spatial heterogeneity of the chemical composition of Rhynie chert fossils, but this technique is unable to identify specific biomarkers [26].

In this study, we describe a novel analytical approach to fossil plant geochemistry which is based on data obtained from whole, intact fossil material using ToF-SIMS and XPS. The two techniques are complementary in that XPS provides information about oxygen-bonded carbon, whereas ToF-SIMS demonstrates the presence of polymeric hydrocarbons. Other advantages of this approach are that there is no requirement to prepare cellulose acetate peels of fossil plant blocks (therefore avoiding deformation to the fragile organic matter during peel preparation [27]) and it is not reliant on a synchrotron X-ray source, which is necessary when studying plant fossil material using X-ray transmission (e.g. [28]). On the other hand, X-ray transmission (carbon (1s) X-ray absorption near-edge spectroscopy (C-XANES)) has greater spatial resolution, so that, for example, it can be used to analyse individual cells [29]. A crucial step in our approach is to remove surface material soluble in the dichloromethane/methanol solvent mixture during the ‘cleaning’ stage. XPS and argon gas cluster ion beam etching were then used to confirm the absence of detectable contamination on the surface of the specimen as described above in §2(a). The high-resolution C 1s spectra (figure 4) identified the same oxygen-bonded carbon, namely C–O, C=O and O–C=O, as was previously detected in *Aglaophyton* and *Rhynia* [28] as well as *Asteroxylon mackiei* [27] from the Rhynie chert using C-XANES. We demonstrate that there is lateral heterogeneity in the distribution of aromatic and aliphatic carbon across the surface of *R. gwynne-vaughanii*. The centre of the *R. gwynne-vaughanii* shown in the left-hand column of images in figure 5 showed significantly higher secondary ion intensity due to both types of hydrocarbon species compared to the adjacent 0.5 mm square (figure 5) within the *R. gwynne-vaughanii* itself and to the surrounding matrix region. This is consistent with increased relative amounts of both aromatic and aliphatic carbon underlying the xylem of the fossil plant. These correlate with the vascular structures observed in the high-resolution optical image presented in figure 5. Further investigation is needed to elucidate the source of the hydrocarbons we detected. It would be instructive to analyse other fossil plants such as *Asteroxylon* using the same technique.

ToF-SIMS analysis is normally considered to be a purely qualitative technique, but it can be *relatively* quantitative in special cases (e.g. for very similar surfaces and when comparing secondary ion peak areas for species present at very dilute concentrations, certainly less than 0.1% w/w). In the case of the secondary ion maps presented in this work, it is reasonable to equate the carbon signal intensity with the relative amount of material present in the surface region analysed. Both ToF-SIMS and XPS results show that surface science technologies can be used to determine the location of the molecular components of fossil plant cell walls while retaining structural information. These techniques show that powdering of valuable fossil samples is not required and in this sense can be compared with the recent report of a direct extraction-free approach for the analysis of lipids in intact sediment cores using laser desorption ionization coupled to Fourier transform ion cyclotron resonance mass spectrometry [29].

Accurate identification of biopolymers and their diagenetic products requires the development of databases from the analysis of plant-derived authentic standards using ToF-SIMS and XPS. Our study shows that if this is done, imaging ToF-SIMS will be a valuable tool to help understand the distribution of biomolecules and their taphonomic/diagenetic products within the organs and tissues of land plant fossils. Further analyses are now under way in our laboratory to characterize the molecular composition of other early land plants as well as enigmatic fossils to help elucidate how terrestrialization shaped the early evolution of land plant cell wall biochemistry.

## Data accessibility

This article has no additional data.
